# Estrogenic and cytotoxic potentials of compounds isolated from *Millettia macrophylla* Benth (Fabaceae): towards a better understanding of its underlying mechanisms

**DOI:** 10.1186/s12906-016-1385-5

**Published:** 2016-10-26

**Authors:** Stéphane Zingue, Job Tchoumtchoua, Dieudonnée Mireille Ntsa, Louis Pergaud Sandjo, Julia Cisilotto, Chantal Beatrice Magne Nde, Evelyn Winter, Charline Florence Awounfack, Derek Tantoh Ndinteh, Colin Clyne, Dieudonné Njamen, Maria Halabalaki, Tânia Beatriz Creczynski-Pasa

**Affiliations:** 1 Laboratory of Physiology and Natural Products Research, Department of Live and Earth Sciences, Higher Teachers’ Training College, University of Maroua, P.O. Box 55, Maroua, Cameroon; 2 Department of Animal Biology and Physiology, Faculty of Sciences, University of Yaoundé I, P.O. Box 812, Yaoundé, Cameroon; 3 Division of Pharmacognosy and Natural Products Chemistry, School of Pharmacy, University of Athens, Panepistimioupoli Zografou, 15771 Athens, Greece; 4 Department of Pharmaceutical Sciences, Health Sciences Centre, Federal University of Santa Catarina, CEP 88040-900 Florianópolis, Santa Catarina Brazil; 5 Hudson Institute of Medical Research, Clayton, VIC 3168 Australia; 6 Department of Applied Chemistry, Faculty of Sciences, University of Johannesburg, Doornfontein, 2028 South Africa

**Keywords:** *Millettia macrophylla*, Phytoestrogens, Estrogen-dependent cancer, Cytotoxicity, E-screen assay, Uterotrophic assay

## Abstract

**Background:**

*Millettia macrophylla* was previously reported to have estrogenic effects and to prevent postmenopausal osteoporosis in Wistar rats. So, the study deals with the identification of its secondary metabolites and the evaluation of their estrogenicity and cytotoxicity toward tumoural cells. Thus, 13 known compounds were obtained from successive chromatographic columns and identified by NMR data compared to those previously reported.

**Methods:**

In vitro estrogenicity of the isolates and the phenolic fraction (PF) of *M. macrophylla* were performed by E-screen and reporter gene assays, while their cytotoxicity was evaluated by Alamar Blue (resazurin) assay. A 3-days uterotrophic assay and the ability of PF to alleviate hot flushes in ovariectomized adult rats were tested in vivo.

**Results:**

Seven of the 13 secondary metabolites turned to be estrogenic. Only two exhibited cytotoxic effects on MCF-7 and MDA-MB-231 with CC_50_ values of 110 μM and 160 μM, respectively. PF induced a significant (*p* < 0.01) MCF-7 cells proliferation and transactivated both ERα and ERβ in the reported gene assay at 10^−2^ μg/mL. In vivo, PF acted more efficiently than the methanol crude extract, resulting to a significant (*p* < 0.01) increase in the uterine wet weight, uterine protein level, uterine and vaginal epithelial height at the dose of 10 mg/kg BW. In addition, PF reduced the average duration and frequency of hot flushes induced in rat.

**Conclusion:**

These aforementioned results indicate that PF is a good candidate for the preparation of an improved traditional medicine able to alleviate some menopausal complaints such as vaginal dryness and hot flushes.

**Graphical abstract:**

Estrogenic and cytotoxic potentials of compounds isolated from *Millettia macrophylla* Benth. (Fabaceae): towards a better understanding of its underlying mechanism
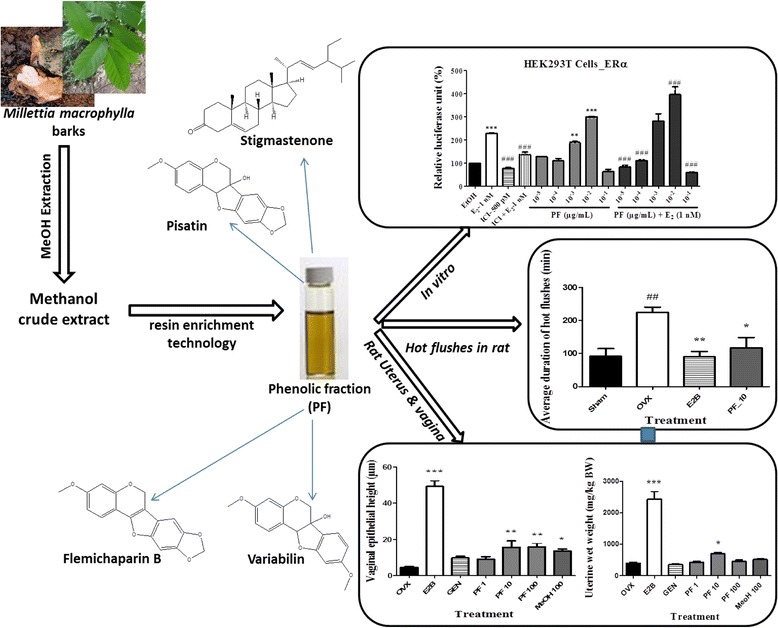

**Electronic supplementary material:**

The online version of this article (doi:10.1186/s12906-016-1385-5) contains supplementary material, which is available to authorized users.

## Background

Cancer is a leading cause of death and a major public concern worldwide. More than 1.1 million people are diagnosed every year with cancer among them 410,000 deaths [1]. In developing countries, gynecological and mammary cancers (breast, uterus, and ovarian) account for 19 % of cancers worldwide and are among the leading causes of morbidity and mortality in these countries [2]. Chemotherapy and surgery remain the major treatments of this ailment although side effects and the treatment expense represent two major difficulties for affected patients, especially those from Sub-Saharan Africans. These two factors make approximately 80 % of the population rely and use medicinal plants for their primary healthcare problems or as an alternative solution to cure diseases [3]. Nowadays, there is a recrudescence of interest for the natural alternative such as medicinal plants and dietary means. Even in developing countries, this phenomenon is observed because of their efficacy and toxicity tolerance [4, 5]. So, medicinal plants are increasingly screened for seeking anticancer hits and numerous phytoconstituents have already been reported to be cytotoxic toward breast tumour cells and to prevent or abrogate tumours induced in rats [6–9]. Phytoestrogens are plant metabolites with the chemical structure shaped as that of 17β-estradiol and mimic estrogenic actions in mammals [10]. Natural estrogenic compounds include coumestans, isoflavonoids and flavonoids [11, 12]. Since they are endowed with both estrogenic and antiestrogenic properties, these natural selective estrogen receptors modulators are now promoted as a preventive alternative against estrogen-dependent cancers such as breast, ovarian, uterine and prostate cancers [13].

As part of our continuous search of new phytoestrogens, we focused the study on *Millettia macrophylla* (Fabaceae), a plant growing in sub-Saharan Africa. It is used for the treatment of respiratory difficulties, constipation, colds and headaches, jaundice as well as some physiological disorders related to menopause [14, 15]. There are also evidences suggesting that plants belonging to the genus Millettia can boost the immune system, and even fight some forms of cancer [16, 17]. Our previous work showed that *M. macrophylla* dichloromethane (DCM) and methanol (MeOH) extracts have estrogen-like effects on estrogen target organs of ovariectomized Wistar rats and prevent postmenopausal osteoporosis [18, 19]. In addition the estrogen-like effects of these extracts were inhibited in vitro and in vivo by the pure ER antagonist ICI 182, 780 (Fulvestrant), indicating that their effects were primarily mediated through ERs [20]. These findings suggest the presence of estrogenic/antiestrogenic compounds in *M. macrophylla* helpful for the treatment of estrogen-dependent cancers, since two thirds of newly diagnosed invasive breast tumors are estrogen-dependent [21]. The present study therefore aimed to identify and isolate the major compounds present in *M. macrophylla* and to assess their estrogenic and cytotoxic effects.

## Methods

### Plant material and extraction

Stem barks of *Millettia macrophylla* were collected in Kumba (South-west Region of Cameroon) in January 2010 and identified at the Cameroon National Herbarium (CNH) (Voucher specimen N°49654/HNC) by the botanist M. Victor Nana. The well-dried and pulverized stem bark of this plant (2,500 g) was extracted successively with DCM and MeOH (10 L of each solvent × 3; 72 h per extraction). Following concentration under reduce pressure, 25 g (1 %) of dichloromethane and 53.4 g (2.14 %) of methanol crude extracts were obtained.

### Isolation of compounds from M. macrophylla DCM extract

Eight grams of DCM extract was chromatographed using a normal phase Medium Pressure Liquid Chromatography (MPLC, BÜCHI Labortechnik AG) conditioned in a column with metal sheath glass (36 × 460 mm), adapted to the pressure of work (15 bar). A glass pre-column was used to reduce the load imposed on the walls of the column. The stationary phase consisted of silica gel 60H (granulometry 20–40 μm). The mobile phase consisted of a binary mixture of solvent of hexane–dichloromethane–ethylacetate–methanol of increasing polarity by step gradient using the pump Büchi C-605, to yield 875 fractions collected and combined on the basis of TLC and HPLC analysis into 40 collective fractions (F1 – F40). The fractions F5 (50–60; 158.6 mg), F10 (123–156; 913.2 mg), F14 (299–353, 328.2 mg) and F18 (439–495, 108 mg) were pure and correspond to compounds 1, 2, 3, and 4, respectively.

A qualitative evaluation of the fractions by TLC and HPLC-PDA (using a gradient method with H_2_O + 2 % acetic acid and CH_3_CN + 2 % acetic acid) was performed in order to select fractions rich in flavonoids and isoflavonoids. Thus, fractions F15 (354–405; 212.4 mg) were subjected to a preparative TLC developed in c-hex/EtOAc: 70/30 (v/v) to give 2. From the fraction F25 (838–850; 44 mg), compound 5 (2.4 mg) was isolated using preparative TLC developed with c-hex/EtOAc: 70/30 (v/v). The F33 (864) and F34 (865) were merged (100 mg) and filtered through a Sephadex LH-20 column (bead size: 25–100 μm). From this fraction, 74 subfractions were obtained. From subfractions 32–41 (24.7 mg), compound 6 (4.1 mg) was obtained after purification on preparative TLC, developed in c-hex/EtOAc: 70/30. Subfractions 42–54 (25 mg) was subjected to preparative TLC using c-hex/EtOAc: 70/30 (v/v) affording to compounds 6 (5.7 mg) and 7 (3.3 mg).

### Isolation of compounds from M. macrophylla phenolic fraction (PF)

In order to maximize phenolic compounds isolation, a *M. macrophylla* phenolic fraction (PF) was prepared using resin enrichment technology. To achieve this goal the Amberlite XAD-7PH resin (Rhom and Hass, France) was used. The resin was conditioned with excess of distilled water and activated in ethanol -EtOH (96.30°) overnight. EtOH was removed by rinsing the activated resin with distilled water. Then, 52 g of *M. macrophylla* MeOH crude extract were diluted in distilled water and sonicated (Elmasonic S100H at 40 °C for 1 h). Further, the obtained solution was subjected to a separating funnel (2,000 L) containing 400 mg of Amberlite XAD-7PH resin, rinsed many times with distilled water and the water-soluble fraction was collected. The phenolics adsorbed by the resin were eluted using EtOH and the obtained phenolic fraction was concentrate in a rotavapor to afford 7.16 g of phenolic fraction (PF).

Six grams of phenolic fraction was chromatographed using a Medium Pressure Liquid Chromatography (MPLC) as described above with the slight difference that the column size was 36 × 460 mm and the applied pressure was 20 bar. The stationary phase consisted of silica gel RP-18 (granulometry 40–63 μm). The mobile phase consisted of a binary mixture of solvent of water–ethanol of increasing polarity by step gradient using the pump Büchi C-605, to yield 693 fractions collected and combined on the basis of TLC and HPLC analysis into 26 collective fractions. Fractions from F9 to F13 (226–380) were merge (240 mg) and subjected to a Sephadex LH-20 gel filtration. Compounds 8 (2 mg) and 9 (1 mg) were isolated using preparative TLC developed with DCM/MeOH (98/02: v/v) of subfractions 77–87 (14.1 mg). Subfractions from 88 to 98 (13.1 mg) were subjected to preparative TLC developed with DCM/MeOH (95/05: v/v) to afford compounds 9 (1 mg) and 10 (0.9 mg). Compound 11 (1.7 mg) was obtained after purification with preparative TLC developed with DCM/MeOH (93/07: v/v) of subfractions 104–116 (12.5 mg). From the initial fractionation, fractions F14 (381–423, 90 mg) and F15 (424–455, 80 mg) were merged and 160 mg were subjected to a Sephadex gel filtration. After a preparative TLC developed with c-hex/EtOAc (70/30: v/v), the subfraction 17–27 (12.3 mg) afford to compounds 10 (2 mg) and 12 (1.8 mg). Subfractions 28–43 (20.6 mg) led to compound 12 (1 mg) after purification on a preparative TLC developed with c-hex/EtOAc (60/40: v/v). The fraction F16 (456–484, 140 mg) was subjected to a silica gel column (granulometry 40–63 μm). Subfractions 28–130 (10.2 mg) were subjected to a preparative TLC developed with c-hex/EtOAc (60/40: v/v) to led compound 10 (1.4 mg). Compound 13 (1.5 mg) was isolated from the subfractions 131–280 (10.3 mg) in the preparative TLC developed with c-hex/EtOAc (40/60: v/v).

### UHPLC-LTQ-Orbitrap Analysis of Millettia macrophylla phenolic fraction

The analysis of the phenolic fraction of *Millettia macrophylla* was performed using an Accela Ultra High-Performance Liquid Chromatography (UHPLC) system equipped with a mixing pump, an autosampler, and hyphenated to a hybrid LTQ-Orbitrap Discovery Mass Spectrometer (Thermo Scientific, Bremen, Germany). Stock solution of 100 μg/mL (MeOH/H2O, 50/50, v/v) of the fraction was prepared and 10 μL injected on a Hypersil Gold column (100 × 2.1 mm i.d., 3 mm) particle size, Thermo Scientific, Waltham, MA). The mobile phase used was aqueous acetic acid 0.1 % (v/v) (solvent A) and acetonitrile (solvent B). The initial conditions were 98 % of solvent A and 2 % of solvent B adjusting linearly to 98 % B in 18 min. This solvent composition was maintained for 0.9 min (98 % B) followed by a return to the initial conditions (in 0.1 min) and a re-equilibration step (1 min) prior to the next run. The flow rate was set to 500 μL/min.

The mass spectrometer was equipped with electro-spray ionization (ESI) source and activated in a positive mode. The analysis was performed in full scan acquisition and the mass tolerance was set to 5 ppm for all measurements. The ESI source was operated at a sheath gas flow rate of 50 arb, auxiliary gas flow rate of 10 arb, ion spray voltage of 3.5 kV, capillary temperature of 300 °C, capillary voltage of 35 V, tube lens of 110 V. Xcalibur 2.0.7 software was used for the pre- and post-acquisition of the results.

### General phytochemistry experimental procedures

A Thermo Finnigan HPLC system (ThermoFinnigan, San Jose, CA) was employed for the profiling of the extract connected to a Spectral System UV2000 PDA detector. ChromQuest 2.1 software was used for the management of the data. Nuclear magnetic resonance (NMR) spectra were obtained on Bruker 600 MHz spectrometer using CDCl3 (Sigma Aldrich, Germany) as solvent. The 2D-NMR experiments (COSY, LRCOSY, HMQC, HSQC-DEPT135, and HMBC) were performed using standard Bruker microprograms. ESI-HRMS were run on a Thermo Scientific LTQ Orbitrap Discovery mass spectrometer. GC-MS analyses were carried out using a Hewlett-Packard 5973–6890 GC-MS system operating in the EI mode at 70 eV, equipped with an HP-5 MS capillary silica column (30 m × 0.25 mm i.d.; 0.25 μm film thickness). Sephadex LH-20 (Merck, Germany) was used as gel filtrate. Precoated TLC silica 60 F254 plates (Sigma Aldrich, Germany) were used for thin-layer chromatography (0.25 and 2 mm layer thickness for analytical and preparative TLC, respectively). Spots were visualized using UV light and vanillin–sulfuric acid reagent.

### Chemicals

Serum and antibiotics were purchased from GIBCO (Grand Island, NY). The 17β-estradiol benzoate (Estr-1,3,5(10)-trien-3,16α,17β-triol) was obtained from Sigma-Aldrich (Hamburg, Germany). The 2-[4-(2-hydroxyethyl)piperazin-1-yl]ethane sulfonic acid (HEPES) was purchased from Ludwig Biotecnologia Ltda (Alvorada, RS, Brazil). Trypan blue, Sulforodamine B, Alamar blue and cell culture mediums were purchased from Sigma-Aldrich (St. Louis, MO, USA). Genistein was obtained from “Extrasynthese®” (Genay, France). Penicillin (xtapen®) was provided by CSPC Zhongnuo pharmaceutical (Shijiazhuang City, China). Diclofenac (Dicloecnu®) was provided by ECNU pharmaceutical (Yanzhou City, China). The Smart Button Data loggers were purchased from ACR System Inc (Surrey, Canada).

### Experimental organisms

#### Cell line systems

The MCF-7 – Human ER-positive breast adenocarcinoma cells and the MDA-MB-231– human ER-negative breast adenocarcinoma cells were obtained from the Rio de Janeiro Cell Bank (Federal University of Rio de Janeiro, Brazil).

HEK293T – Human Embryonic Kidney 293 T cells line that contain the SV40 large T-antigen were purchased from ATCC (The Global Bioresource Center, Australia). Luciferase reporter construct, ERα and ERβ expression plasmids were kindly provided by Dr Simon Chu (Hudson Institute of Medical Research, Australia). Cells were transfected using Lipofectamine Reagent obtained from Invitrogen (Sydney, Australia).

#### Animals

Healthy juvenile female Wistar rats aged 3 months (~150g) were obtained from the breeding facility of the Laboratory of Animal Physiology, University of Yaounde I (Cameroon). Animals were housed in clean plastic cages at room temperature (around 25 °C) under natural illumination (approx. 12 h light/dark). They had free access to a standard soy-free rat chow and water *ad libitum*. The composition of animal diet was: corn (36.7 %), bone flour (14.5 %), wheat (36.6 %), fish flour (4.8 %), crushed palm kernel (7.3 %), sodium chloride (0.3 %) and vitamin complex (Olivitazol® - 0.01 %).

#### Ethical consideration

Housing of animals and all experiments were approved by the Cameroon Institutional National Ethic Committee, which adopted all procedures recommended by the European Union on the protection of animals used for scientific purposes (CEE Council 86/609; Reg. no. FWA-IRD 0001954).

### In vitro experiments

#### Cell culture

MDA-MB-231 cells were cultured in DMEM medium supplemented with 10 % of fetal bovine serum (FBS). MCF-7 cells were cultured in RPMI-1640 medium supplemented with 10 % of FBS. HEK293T cells were cultured in DMEM medium supplemented with 10 % fetal calf serum (FCS). All cell cultures were also supplemented with 100 U/mL penicillin, 100 μg/mL streptomycin and 10 mM HEPES. The cell cultures were maintained at 37 °C in a 5 % CO_2_ humidified atmosphere and pH 7.4. Every two days, cells were passaged by removing 90 % of the supernatant and replacing it with fresh medium. In all experiments, viable cells were checked at the beginning of the experiment by Trypan Blue dye exclusion test.

#### Cell viability assay

Cytotoxicity potentials of isolates from *M. macrophylla* as well as phenolic fraction were evaluated by Alamar Blue (resazurin) assay [22], in two independent cellular systems (MCF-7 and MDA-MB-231). To evaluate the influence of concentration and time on cytotoxicity, 1 × 10^4^ cells/well were seeded in a 96-well plate in 100 μL of culture medium. After 24 h to permit their adhesion, cells were exposed to the 13 compounds isolated from *M. macrophylla* (1 to 13) at concentrations ranging from 100 to 500 μM. *M. macrophylla* phenolic fraction (PF) was also tested at concentrations of 100 and 500 μg/mL for 24 h. The CC_50_ value (cytotoxic concentration, which means concentration required to reduce the cell number by 50 %) was determined only with promising compound by nonlinear regression analysis of the logarithm of concentration in function of the normalized response (percentage of cell viability) using the software GraphPad Prism 5.0. Each experiment was performed in at least triplicate and repeated three times.

#### E-screen assay

In order to evaluate estrogenic-like effects of isolates from *M. macrophylla* a simple and sensitive E-screen cell proliferation assay was performed with human ER-positive breast adenocarcinoma cells (MCF-7). This assay determines the estrogenicity/antiestrogenicity of compounds indirectly through measurement of the proliferation of MCF-7 cells. For this purpose, the technique slightly modified by Resende et al. [23], from the originally described by Soto et al. [24] was used. Briefly, cells were trypsinized and seeded in 24-well plates at an initial concentration of 20,000 cells per well in 10 % FBS in RPMI. After 24 h of incubation (37 °C, 5 % CO_2_) to permit their adhesion, cells were washed with phosphate-buffered saline (PBS) and the Serum Replacement 2 (0.5×) supplemented phenol red-free RPMI was substituted for the seeding medium. *M. macrophylla* compounds were added to the experimental medium at concentrations from 0.1 to 10 μM. For antiestrogenicity tests, before incubation, 1 × 10^−8^ M of 17β-estradiol was added to the wells. Steroid-free experimental medium consisted to negative control while cells treated with 1 × 10^−8^ M of 17β-estradiol served as positive control. There were also a solvent control (DMSO at 0.01 %) and a medium control (10 % FBS in RPMI). The assay was stopped after 144 h by removing the medium from wells, fixing the cells, and staining them with sulforhodamine-B (SRB). Briefly, cells were treated with cold 10 % trichloracetic acid and incubated at 4 °C for 1 h. Then, the cells were washed four times with tap water and dried. Furthermore, cells were stained during 30 min with 0.057 % (w/v) SRB dissolved in 1 % acetic acid. Wells were rinsed four times with 1 % acetic acid and air dried. Bound dye was solubilized with 10 mM Tris base (pH 10.5) in a shaker. Finally, aliquots were read in a Biotek EL800 Multiscan apparatus (Winoosky, USA) at 510 nm.

The results expressing the estrogenic activity were showed as mean ± standard error of mean of the proliferative effect (PE), which represents the maximum proliferation induced by the compounds. This parameter was calculated according to Schiliro’et al. [25], and is the ratio between the highest cell number achieved with the sample or 17β-estradiol and the cell number in the solvent control (0.01 % DMSO): *PE = max cell number of sample/cell number of DMSO control*.

The estrogenic activity of a sample was determined as the relative proliferative effect (RPE%). The RPE compares the maximum proliferation induced by a sample with that induced by 17β-estradiol: *RPE% = [PE for sample/PE for 17β-estradiol]* × *100* [23].

#### Transfections and luciferase assays

Due to the available quantity of isolates of *M. macrophylla*, only stigmastenone (3) and phenolic fraction (PF) has been deal with their capacity to activate estrogen receptors α and β, in cell-reporter gene assays. The Human Embryonic Kidney 293 T cell line (HEK293T) was transiently transfected with adequate plasmids using Lipofectamine Reagent as previously described by Zingue et al. [18]. They were then treated with different concentrations [10^−9^ to 10^−5^ M for stigmastenone (3) and 10^−5^ to 10^−1^ μg/mL for phenolic fraction] for 24 h. Cells treated with E2 (10 nM) alone served as positive control. Luciferase activity was measured and normalised against β-galactosidase activity as previously described [18].

### In vivo experiments

#### The 3-day uterotrophic assay

To achieve this goal, 35 female Wistar rats received a single intramuscular dose of long acting penicillin and diclofenac (10 mg/kg and 3 mg/kg respectively) the day before ovariectomy. Thereafter they were bilaterally ovariectomized (OVX) using the dorsal approach [26] under Diazepam and ketamin anesthesia (respectively 10 mg/kg and 50 mg/kg BW; i.p.). After 14 days of endogenous hormonal decline, animals were randomly distributed into seven groups of five animals each (n = 5). The first group or OVX group received vehicle only (corn oil) and the second group received estradiol Benzoate (E2B) as standard drug at the optimal dose of 2 μg/kg per day. The third group received genistein at the dose of 10 mg/kg. The remaining four groups received either the phenolic fraction (PF) at doses of 1, 10 and 100 mg/kg or the methanol crude extract (MeOH) of *M. macrophylla* at dose of 100 mg/kg. All treatments were administered by subcutaneous route (0.3 mL/150 g) for 3 days. Twenty-four hours after the last administration, animals were euthanized by decapitation. The uterine wet weight, total protein levels in uterine, uterine and vaginal epithelial heights and mammary gland were assessed as described before by Zingue et al. [20].

#### Measurement of hot flushes

Data loggers were used to monitor the core temperature changes in the animals at 2 min intervals for 72 h, as previously described by Zingue et al. [27]. In this study, data loggers were preset to start measuring core temperatures 12 h before the beginning of the treatment until the end of treatment. Twenty acclimatized rats were either sham-operated (Sham) or bilaterally ovariectomized (OVX) as described above, and at the same time underwent the implantation of a data logger protected in sterilized neutral wax into their abdominal cavities. After 14 days of endogenous hormonal decline, animals were randomly distributed into four groups of five rats each (*n* = 5). They were treated for 3 days with corn oil as vehicle (OVX and Sham groups), estradiol benzoate (E2B) (2 μg/kg BW per day), and the active dose of the phenolic fraction (10 mg/kg BW per day). All treatment was given by subcutaneous route (0.3 mL/150 g) and lasted 3 days. Twenty four hours after the last administration, animals were euthanized by decapitation, and the data loggers recovered. Data (central body temperature) was retrieved from loggers into excel spreadsheets and analyzed using the ACR Trend Reader for Smart Button Software. Hot flushes were considered for any internal temperatures ≥ 38 °C. The total number of hot flushes, the average of these hot flush durations and the frequency of hot flushes were determined as described before [27].

### Histological analysis

Using the complete Zeiss equipment consisting of a microscope Axioskop 40 connected to a computer where the image was transferred, and analyzed with the MRGrab1.0 and Axio Vision 3.1 softwares, all provided by Zeiss (Hallbermoos, Germany), the histomorphology of the mammary glands, as well as the uterine and vaginal epithelial heights, were assessed from 5-μm sections of paraffin-embedded tissues following hematoxylin-eosin staining.

### Biochemical analysis

Total uterine protein levels were determined in uteri using colorimetric methods described by Gonal et al. [28].

### Statistical analysis

Results are presented as means ± standard error of mean (SEM). In vitro experiments were performed in triplicates and repeated three times. All formulas and functions were calculated with Microsoft Excel software. Data analysis was performed with GraphPad Prism 5.0 software, using the ANOVA test followed by the Dunnett’s post hoc test. Differences were considered significant at a probability level of 5 % (*p* < 0.05).

## Results

### Phytochemical analysis of M. macrophylla

#### Identification of M. macrophylla isolates

**Fig. 1:**
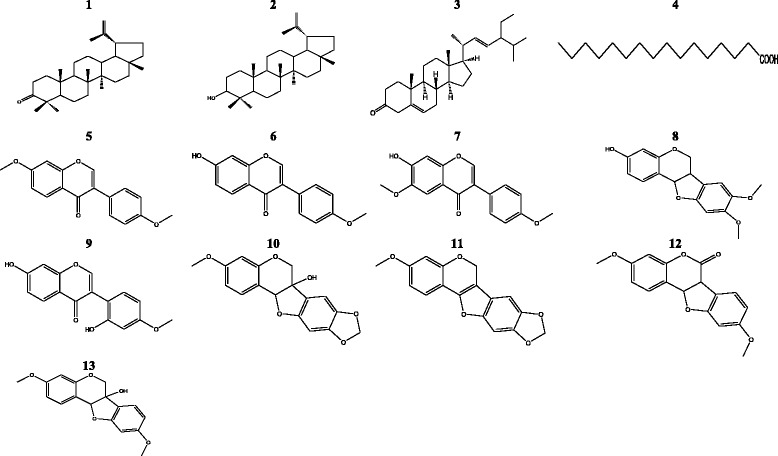
Structure of compounds isolated from *M. macrophylla*

A total of 13 secondary metabolites were isolated from *M. macrophylla* stem barks extracts. The structures of 1, 2, and 5–13 were elucidated by 1D & 2D NMR as well as HRMS and by comparison to previously reported data (Additional file 1). Compounds 3 and 4 structures were determined by GC-MS. The *M. macrophylla* isolates (Fig. 1) were identified as lupenone (1) [29], lupeol (2) [30], stigmastenone (3) [31], palmitic acid (4) [32], daidzein dimethylether (5) [33], formononetin (6) [34], afromorsin (7) [34], secundiferol I (8) [35], 2′-hydroxyformononetin (9) [36], pisatin (10) [37], flemichapparin B (11) [38], dihydrocoumestrol dimethyl ether (12) [39] and variabilin (13) [40].

#### UHPLC-LTQ-Orbitrap Analysis of Millettia macrophylla phenolic fraction

**Fig. 2:**
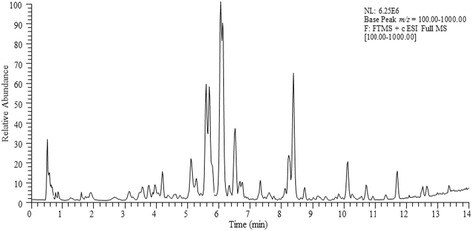
Base peak chromatogram of *Millettia macrophylla* phenolic fraction recorded in UHPLC-ESI (+)-LTQ-Orbitrap

The profiling of the phenolic fraction of *Millettia macrophylla* revealed the presence of the several secondary metabolites covering a wide polarity range (Fig. 2). Amongst others, 12 compounds could be tentatively identified. Chromatographic and spectrometric features such as retention time (Rt), high resolution quasi-molecular ion (HRMS, *m/z*), suggested elemental composition, Ring Double Bond equivalent (RDBeq) were incorporated to the identification procedure. Specifically, six isoflavonoids (daidzein, daidzein dimethyl ether, biochanin A, 7-methyl tectorigenin, formononetin, afromorsin), three pterocarpans (maackiain, pisatin, flemichapparin B), one coumestan (coumestrol methyl ether), and one simple phenolic (2,4,6-Trimethoxyphenol) could be detected (Table 1). However, three compounds initially isolated from the phenolic fraction namely secundiflorol I, glycinol dimethyl ether, and dihydro-coumestrol dimethyl ether were not identified in the UHPLC-MS profile probably due to their minor concentration in the whole fraction.

**Table 1:** Chromatographic and spectrometric characteristics of secondary metabolites identified in *Millettia macrophylla* phenolic fraction using UHPLC-ESI (+)-LTQ-Orbitrap

Peak	Rt (min)	Experimental *m/z*	Theoretical *m/z*	Δ (ppm)	RDB	[M + H]^+^	Assignment
1	1.61	185.0804	185.0808	−2.352	3.5	C_9_H_13_O_4_	*2,4,6-Trimethoxyphenol*
2	2.81	255.0646	255.0652	−2.452	10.5	C_15_H_11_O_4_	*Daidzein*
3	3.97	285.0750	285.0757	−2.491	10.5	C_16_H_13_O_5_	*Maackiain*
4	6.53	285.0753	285.0757	−1.614	10.5	C_16_H_13_O_5_	*Biochanin A*
5	6.69	315.0856	315.0863	−2.173	10.5	C_17_H_15_O_6_	*Pisatin*
6	7.68	315.0855	315.0863	−2.649	10.5	C_17_H_15_O_6_	*7-Methyl tectorigenin*
7	8.24	269.0801	269.0808	−2.696	10.5	C_16_H_13_O_4_	*Formononetin*
8	8.39	299.0908	299.0965	−1.939	10.5	C_17_H_15_O_5_	*Afromorsin*
9	8.75	297.0751	297.0757	−2.289	11.5	C_17_H_13_O_5_	*Flemichapparin B*
10	10.12	283.0957	283.0965	−2.775	10.5	C_17_H_15_O_4_	*Daidzein dimethyl ether*
11	10.70	283.0594	283.0601	−2.508	11.5	C_16_H_11_O_5_	*Coumestrol methyl ether*

### Effects of M. macrophylla isolates on cell viability

The cytotoxic potential of *M. macrophylla* isolates is depicted in Table 2. No cytotoxic effects were found with compounds 1, 3–5, 9–12 up to 500 μM in both MCF-7 and MDA-MB-231 cells. However, only 2 (110 μM) and PF (452 μg/mL) induced a reduction of 50 % of MCF-7 cells viability. MDA-MB-231 cells were more sensitive to the isolates than MCF-7 cells. CC_50_ values of 2, 6, 7, 8, 13 and PF against MDA-MB-231 cells were 160 μM, 342 μM, 224 μM, 457 μM, 296 μM, and 327 μg/mL, respectively.

**Table 2:** Cytotoxicity of isolates of *M. macrophylla* as well as phenolic fraction toward breast cancer cells

		CC50	
Code	Name of compounds	MCF-7 cells	MDA-MB-231 cells
1	lupenone	>500	>500
2	lupeol	**110**	**160**
3	stigmastenone	>500	>500
4	palmitic acid	>500	>500
5	daidzein dimethylether	>500	>500
6	formononetin	>500	**342**
7	afromorsin	>500	**224**
8	secundiferol I	>500	**457**
9 2’	OH-formononetin	>500	>500
10	(+)-pisatin	>500	>500
11	flemichapparin B	>500	>500
12	dihydrocoumestrol dimethylether	>500	>500
13	variabilin	>500	**296**
PF	phenolic fraction	**452**	**327**

MCF-7 and MDA-MB-231 cells were incubated with increasing concentration (100–500 μM) of isolates of *M. macrophylla* and phenolic fraction -PF (100–500 μg/mL) for 24 h. Cell viability was evaluated by Alamar blue assay. The CC50 was determined when applicable by nonlinear regression analysis of the logarithm of concentration in function of the normalized response (percentage of cell viability). Values are in μM or μg/mL mean of three independent experiments. The Bold data reflect the exact cytotoxic concentration (CC_50_)

### In vitro estrogenic effects of M. macrophylla isolates

#### E-screen assay

**Fig. 3:**
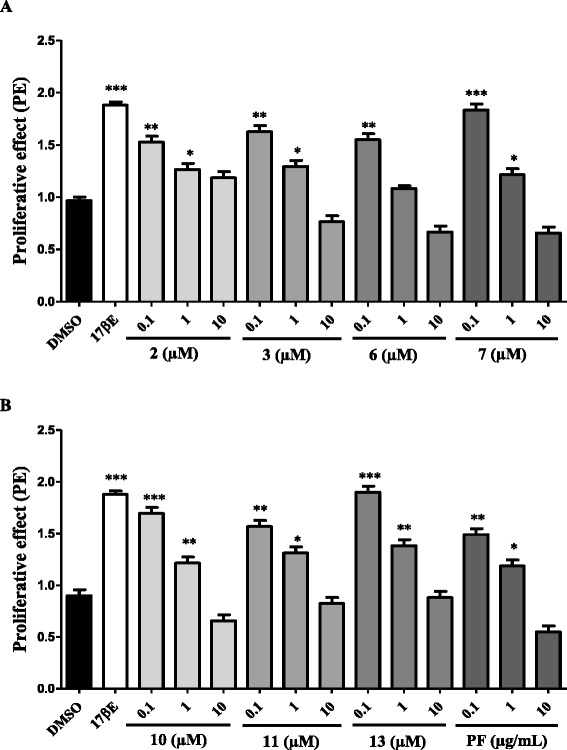
Effects of lupeol (2), stigmastenone (3), formononetin (6), afromorsin (7) (**a**) and of pisatin (10), flemichapparin B (11), variabilin (13), phenolic fraction (PF) (**b**) from *M. macrophylla* on MCF-7 cells proliferation. The effect of compounds and phenolic fraction was investigated by measuring E-screen assay. The relative MCF-7 cells yields (PE) were measured in the presence of DMSO (0.01 %), 17β-estradiol (10 nM), compounds and phenolic fraction from *M. macrophylla*. PE = max cell number of sample/cell number of DMSO control. * *p* < 0.05, ** *p* < 0.01, *** *p* < 0.001 as compared with DMSO

Results of the in vitro estrogenic potencies of *M. macrophylla* isolates are illustrated in Table 3 and Fig. 3. Lupeol (2), stigmastenone (3), formononetin (6), afromorsin (7), pisatin (10), flemichaparin B (11), variabilin (13) and phenolic fraction (PF) of *M. macrophylla* induced a significant (*p* < 0.05 to *p* < 0.001) proliferation of MCF-7 cells such as 17β-estradiol (*p* < 0.001) as compared to DMSO control. Moreover, the rank order of the ability of these compounds to induce MCF-7 proliferation was: 13 > 7 > 10 > 3 > 11 > 6 > 2 > phenolic fraction. Although it has been observed decreased of MCF-7 cells yield with increasing concentration of compounds, no significant antiestrogenic effect was observed in this assay (data not shown).

**Table 3:** Effects of isolates of *M. macrophylla* in MCF-7 cells proliferation assay

Code	Name of compounds	Concentration	PE	RPE (%)
DMSO	Control	-	1	54.2
E2B	17β-estradiol	10 nM	1.84	100
1	Lupenone	0.1 μM	1.38	75
		1 μM	1.22	66.48
		10 μM	0.47	25.53
2	lupeol	0.1 μM	1.52	**82.45**
		1 μM	1.26	68.62
		10 μM	1.19	64.36
3	stigmastenone	0.1 μM	1.63	**88.30**
		1 μM	1.29	70.21
		10 μM	0.76	41.49
4	palmitic acid	0.1 μM	1.36	73.94
		1 μM	1.13	61.17
		10 μM	0.65	35.11
5	daidzein dimethylether	0.1 μM	1.29	70.21
		1 μM	1.33	72.34
		10 μM	0.90	48.93
6	formononetin	0.1 μM	1.55	**84.04**
		1 μM	1.05	56.91
		10 μM	0.67	36.17
7	afromorsin	0.1 μM	1.83	**99.47**
		1 μM	1.21	65.96
		10 μM	0.66	35.64
8	secundiferol I	0.1 μM	1.17	63.83
		1 μM	1.33	72.34
		10 μM	0.68	36.70
9	2’ OH-formononetin	0.1 μM	1.08	58.51
		1 μM	0.99	53.72
		10 μM	0.65	35.11
10	pisatin	0.1 μM	1.70	**92.02**
		1 μM	1.21	65.96
		10 μM	0.66	35.64
11	flemichapparin B	0.1 μM	1.57	**85.11**
		1 μM	1.31	71.28
		10 μM	0.82	44.68
12	dihydrocoumestrol DME	0.1 μM	1.23	67.02
		1 μM	1.16	62.76
		10 μM	0.81	44.15
13	glycinol dimethylether	0.1 μM	2	**108.51**
		1 μM	1.38	75
		10 μM	0.88	47.87
PF	Phenolic fraction	0.1 μg/mL	1.49	**80.85**
		1 μg/mL	1.17	64.36
		10 μg/mL	0.55	29.78

*DMSO* negative control, *E2B* positive control, *PE* proliferative effect calculated as the effect on solvent control, *RPE* relative proliferative effect, compares the maximum proliferation induced by a sample with that induced by 17β-estradiol. The Bold data represents the values considerate as estrogenic

#### Luciferase assay

**Fig. 4:**
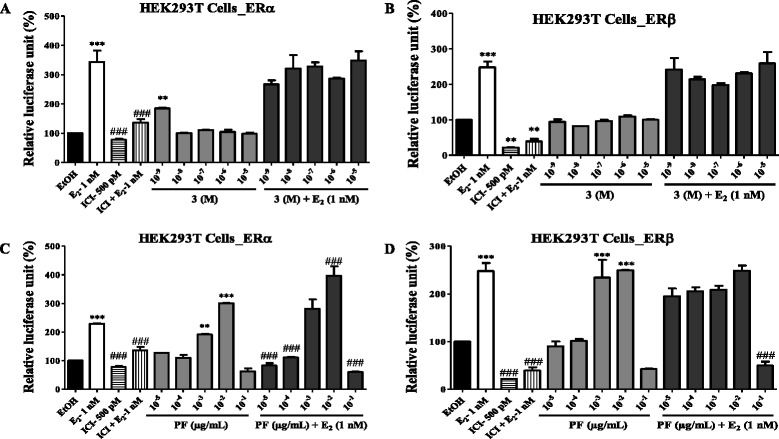
Effects of stigmastenone (3) and *M. macrophylla* phenolic fraction (PF) on the activation of estrogen α and β receptors in HEK293T cells. The effect of these substances on estrogen α and β receptors activity in the transiently transfected HEK293T-ERα and HEK293T-ERβ cells was investigated by measuring reporter gene-coupled luciferase activity. The relative luciferase units (RLU) were measured in the presence of EtOH (0.1 %), E2 (10 nM), stigmatenone (3) as well as *M. macrophylla* phenolic fraction co-treated with E2. ** *p* < 0.01; *** *p* < 0.001 as compared to EtOH control; ### *p* < 0.001 as compared with E2 control

It was found in this work that stigmastenone (3) significantly (*p* < 0.01) transactivated the ERα only at the concentration of 10^−9^ M and this transactivation seems to decrease while its concentration rises up (Fig. 4a). In other hand, 3 failed to transactivated or antagonized the estrogen activity on ERβ (Fig. 4b). As far as *M. macrophylla* phenolic fraction is concerned, it induced a significant transactivation of the ERα as well as the ERβ at concentrations of 10^−3^ (*p* < 0.01) and 10^−2^ μg/mL (*p* < 0.001), while this effect disappear at the concentration of 10^−1^ μg/mL (Fig. 4c). Moreover, the phenolic fraction of *M. macrophylla* seems to be antagonistic to E2 at the lower (10^−5^ and 10^−4^ μg/mL) and higher (10^−1^ μg/mL) concentrations, while it exhibited a synergistic activity with E2 at the concentration of 10^−2^ μg/mL in HEK293T-ERα. A significant (*p* < 0.001) antiestrogenic activity was also observed with the phenolic fraction of *M. macrophylla* on HEK293T-ERβ at the concentration of 10^−2^ μg/mL (Fig. 4d).

### In vivo estrogenic effects of M. macrophylla phenolic fraction

#### Effects on uterus

**Fig. 5:**
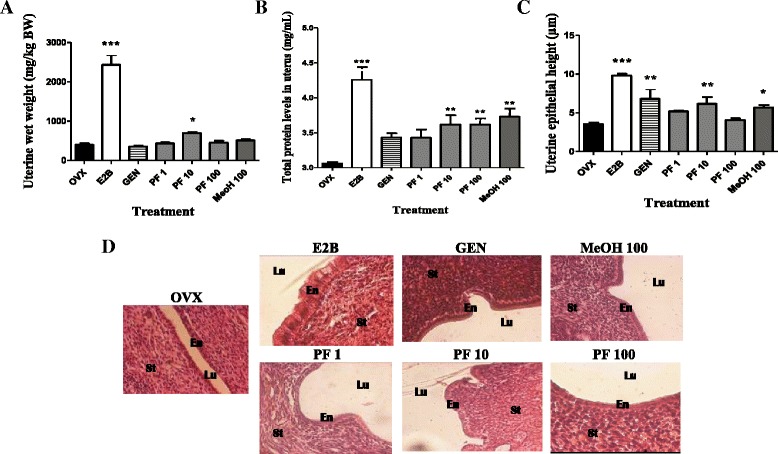
Effects of a 3-day treatment with *M. macrophylla* phenolic fraction on the uterine wet weight (**a**), total protein levels in uterine (**b**), uterine epithelial height (**c**) and microphotographs (**d**). OVX = OVX animals treated with the vehicle; E2B = OVX animals treated with estradiol benzoate at 2 μg/kg BW; PF = OVX animals treated with the *Millettia macrophylla* phenolic extract; MeOH = OVX animals treated with the methanol extract of *M. macrophylla*. *p < 0.05, **p < 0.01; ***p < 0.001 as compared with OVX control. Lu: uterine lumen; En: Endometrium; St: Stroma

Figure 5 showed that E2B induced a significant increase (*p* < 0.001) in the uterine wet weight (Fig. 5a) as well as total protein levels in the uterus (Fig. 5b) as compared to the OVX group. Genistein at the dose of 10 mg/kg did not increase the uterine wet weight while it induced a non-significant increase in uterine total protein levels. The phenolic fraction led to a significant increase (*p* < 0.05) in the uterine wet weight (693.31 ± 52.04 vs 399.31 ± 31.12 mg/kg in OVX group) only at the dose of 10 mg/kg and in the uterine total protein level at the dose of 10 (4.0 ± 0.54 vs 3.1 ± 0.02 mg/mL in OVX group) and 100 mg/kg (3.6 ± 0.08 vs 3.1 ± 0.02 mg/mL in OVX group), while the methanol crude extract did not changes the uterine wet weight.

Figure 5d represents the micrographs of the uterus following 3 days of treatment with different substances. It can be observed that the uterine epithelium of OVX animals consisted to one layer of very flattened cuboidal cells while, animals treated for 3 days with E2B present epithelium composed with large columnar cells. The measurements of the uterine epithelia height showed that the E2B and genistein induced a significant increase (*p* < 0.001 and *p* < 0.01) (Fig. 5c) as compared to the OVX group. *M. macrophylla* phenolic fraction has increased uterine epithelium height (6.15 ± 0.87 vs 3.54 ± 0.21 μm; *p* < 0.01) at the dose of 10 mg/kg more than methanol extract at the dose of 100 mg/kg (5.68 ± 0.32 vs 3.54 ± 0.21 μm, *p* < 0.05) as compared to the OVX group.

#### Effects on vagina

**Fig. 6:**
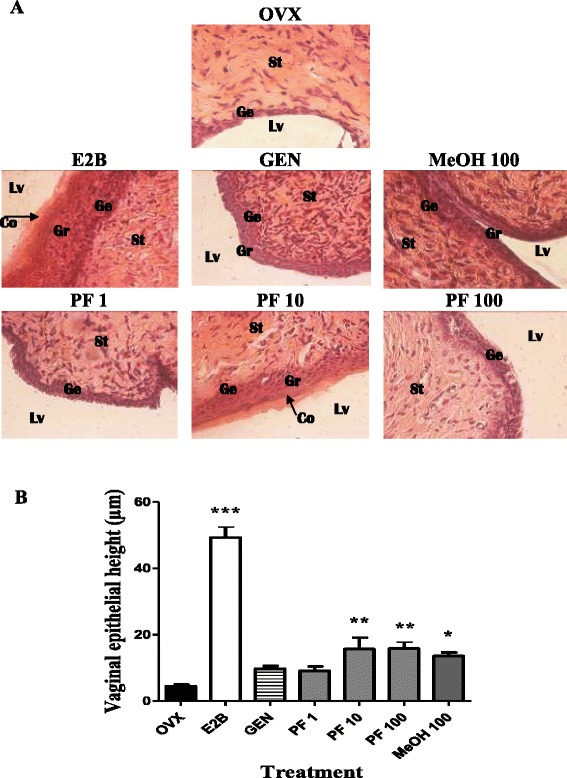
Effects of a 3-day treatment with *Millettia macrophylla* phenolic fraction on the vaginal epithelium: microphotographs (**a**) and epithelial height (**b**). OVX = OVX animals treated with the vehicle; E2B = OVX animals treated with estradiol benzoate at 2 μg/kg BW; PF = OVX animals treated with *Millettia macrophylla* phenolic fraction; MeOH = OVX animals treated with the methanol extract of *M. macrophylla*. *p < 0.05, **p < 0.01, ***p < 0.01 as compared with control. Lv = vaginal lumen, Co = stratum corneum, Gr = stratum granulosum, Ge = stratum germinativum, St: Stroma

Analysis of vagina’s microphotographs from different treatment groups are shown in Fig. 6a. The vagina epithelium of OVX animals is limited to the germinal layer (Ge), consisting of 5–6 cell layers, whereas the vaginal epithelium of animals treated for 3 days with E2B became stratified. Similar stratification coupled with vaginal cornification was observed also in animals treated with the phenolic fraction at the dose of 10 mg/kg (*p* < 0.01). Furthermore, the measurement of vaginal epithelial height in different treated groups showed that the E2B induced a significant increase (*p* < 0.001) in the vaginal epithelial height as compared to the OVX group (Fig. 6b). A significant increase in the vaginal epithelium height was observed after administrating PF at doses of 10 and 100 mg/kg as well as the methanol extract of *M. macrophylla* at the dose of 100 mg/kg. However it can be noted that the increment induced by phenolic fraction (15.63 ± 3.46 vs 4.47 ± 0.51 μm, *p* < 0.01) was greater than that observed with the methanol extract (13.61 ± 1.02 vs 4.47 ± 0.51 μm, *p* <0.05) as compared to the OVX group.

#### Effects on mammary gland

**Fig. 7:**
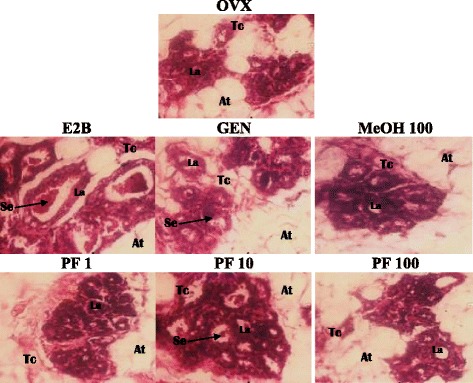
Effects of a 3-day treatment with *M. macrophylla* extracts on mammary gland. OVX = OVX animals treated with the corn oil; E2B = OVX animals treated with estradiol benzoate 2 μg/kg BW; PF = OVX animals treated with the *Millettia macrophylla* phenolic fraction; MeOH = OVX animals treated with the methanol extract of *M. macrophylla*. La = lumen of alveoli; Ep = aveoli epitheluim; At = adipose tissue; Se = eosinophil secretion

The microphotographs of mammary glands (Fig. 7) revealed that OVX animals exhibited smaller acini with non-differentiate lumen of acini, low connective parenchyma while, adipose tissue is abundant. The E2B treatment induced an increase in the size of acini as well as the lumen of acini, which appear well differentiated compared to the OVX group. It can be also observed the presence of abundant eosinophil secretions in the lumen of the acini. The treatment with genistein induced such effects although the arising of the eosinophil secretions in the lumen of acini was low. As far as phenolic fraction and methanol extract of *M. macrophylla* are concerned, they induced an increase in the lumen of the acini at all tested doses as compared to OVX group. However eosinophil secretions were observed only in animal’s group treated with phenolic fraction at the dose of 10 mg/kg.

### Effects on hot flushes

**Fig. 8:**
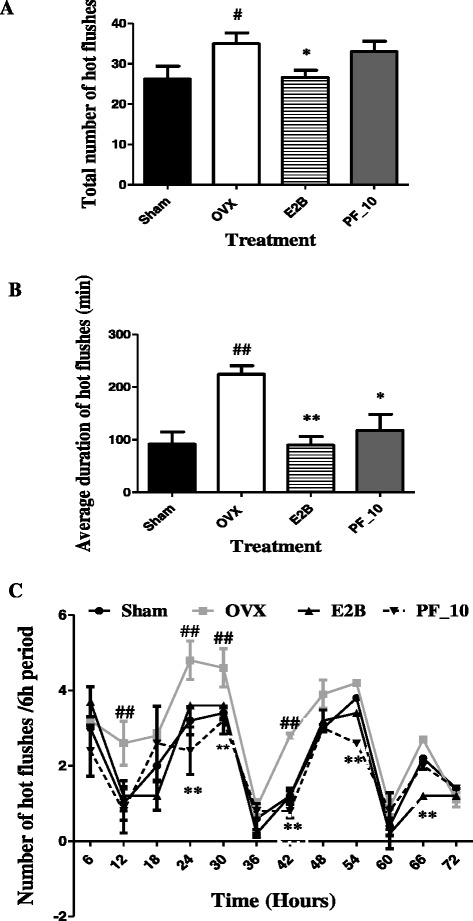
Effects of a 3-day treatment with *Millettia macrophylla* phenolic fraction on total number (**a**), average duration (**b**) and frequency (**c**) of hot flushes. Sham = Sham operated rats treated with the vehicle (corn oil); OVX = OVX animals treated with the vehicle (corn oil); E2B = OVX animals treated with estradiol benzoate at 2 μg/kg BW; PF = OVX animals treated with *Millettia macrophylla* phenolic fraction. * *p* < 0.05, ** *p* < 0.01 as compared to control. ^#^ *p* < 0.05, ^##^ *p* < 0.01 as compared to Sham

Two weeks after oophorectomy, the total number of hot flushes (*p* < 0.05, Fig. 8a) as well as the average duration of hot flushes (*p* < 0.01; Fig. 8b) were significantly increased in the OVX group compared to the normal group (Sham). A significant decrease in the number (*p* < 0.05) and in the average duration (*p* < 0.01) of hot flushes was observed in the E2B treated group. After 3 days of treatment, the phenolic fraction at the dose of 10 mg/kg induced a significant decrease (*p* < 0.05) in the average duration of hot flushes while, it failed to reduce the total number of hot flushes. As compared to the normal group (Sham), OVX animals showed a significant increase (*p* < 0.01) in the frequency of hot flushes (Fig. 8c). As expected, E2B induced a significant decrease (*p* < 0.01) in the frequency of hot flushes as compared to OVX group. Such effects were also observed with phenolic fraction of *M. macrophylla* at a dose of 10 mg/kg (*p* < 0.01).

## Discussion

In order to contribute to a better understanding of the previous reported estrogenic/antiestrogenic properties of *M. macrophylla* [18–20], its major components were isolated. As striking observation, it was noted that most of these compounds belonging to the flavonoids group, specifically, isoflavone, pterocarpan and coumestan subgroups, which are well known as phytoestrogens.

Although the uterotrophic assay in rodents is a good tool to assess estrogenic potency of xenobiotics, this method is not suitable for the screening of large suspected estrogenic chemicals [41]. We have therefore used the biologically equivalent E-screen assay described by Soto et al. [24] and modified by Resende et al. [23]. This assay compares the cell yield between cultures of MCF-7 cells treated with estradiol and cultures treated with different concentrations of substances suspected to endow estrogenic properties. It was noted in this study that 7 out of 13 isolated compounds from *M. macrophylla* induced MCF-7 cells proliferation such as 17β-estradiol in the following rank order: 13 > 7 > 10 > 3 > 11 > 6 > 2 > phenolic fraction. The E-screen assay measures the direct cell proliferation, which is acknowledged as hallmark of estrogenicity [24]. Indeed, the MCF-7 cells proliferation is the reliable estrogenic potency because this cell line is ER positive and it expresses aromatase and 5 α-reductase enzymes, which allows it to convert androgens to estrogens [23, 41]. These authors reported that a relative proliferative effect (RPE) ≥ 80 corresponds to estrogenic activity and suggest that the compound may have agonistic activity to ERα. However, it is worthwhile to highlight that the MCF-7 cells can elicit an estrogen-induced response involving both genomic and non-genomic pathways [25]. Hence, such as in in vivo condition, the MCF-7 cells proliferation observed in E-screen assay is not limited to the activation of ERs [23].

It is well documented that some chemical features in the structure of flavonoids are required to obtain an estrogenic response [42]. A particular note was aroused on the hydroxyl group at position 4′ which promotes the estrogenic activity and the estrogenic potency [43]. Accordingly, the lack of free hydroxyl group at position 4′ or 7′ in 5 might explain its weak affinity for ERs. Indeed, Khan et al. [44] showed that 3,4′,-dimethoxy daidzein had no estrogenicity. This result is consistent with the observation made in this work with 5. However, they reported that it endow positive skeletal effects in osteopenic rats. This might account for the preventive effect previously observed with *M. macrophylla* in postmenopausal osteoporosis [19]. The estrogenic activity of *M. macrophylla* could be explained in part by the presence of 6, which activates the expression of the estrogen-responsive reporter gene in human breast cell line MCF-7 in a concentration-dependent manner (0.5–500 μM). Moreover, this activation was inhibited by estrogen antagonist (ICI 182, 780 at 100 nM) [45]. Furthermore, authors have reported that formononetin induced the proliferation of MCF-7 breast cancer cells, which is consistent with our observations. Other compounds that might contribute to the estrogenicity of *M. macrophylla* are 13 and 10. In fact, 10 has been reported to have a weak affinity to ERα [46] while glycinol an analogue of 13 found in *M. macrophylla* displayed estrogenic effect by induction of MCF-7 cells proliferation at concentration ranging from 1 to 10 μM [47]. These results are consistent with the obtained results with variabilin in this study. In addition, authors showed that glycinol has a high affinity for both ERα (IC_50_ = 13.8 nM) and ERβ (IC_50_ = 9.1 nM). 11, 3 and 2 are described for the first time as estrogenic in this work. 11 is structure related to 10, this can explain its estrogenic activity. As far as stigmastenone and lupeol are concerned, they are triterpernoids, and there is little reports in the literature dealing the estrogenic activity of triterpernoids. However, triterpernoid glycosyl such as glycyrrhizin and ginsenoside-Rh2 have been reported to endow estrogenic properties [48]. Indeed, ginsenoside-Rh2 has been shown to transactivate the ERα in MCF-7, and its effect was abrogated by the pure antiestrogen faslodex (ICI 182,780), suggesting that its effects are primarily mediated by ERs [49]. It was observed a decrease in the proliferative effect of compounds with increasing concentrations (from 0.1 to 10 μM), while they did not induce cytotoxicity at concentration 10 fold higher, avoiding the hypothesis of the cytotoxicity at these concentrations. However, the results obtained with 3 in MCF-7 cells proliferation assay are in accordance with those observed in the reporter gene assay. In fact, such as in MCF-7 cells proliferation, it was noted a concentration-dependent decrease in the transactivation induced by 3 in ERα. This result strongly suggests a down regulation phenomenon rather than a cytotoxicity of these compounds. This phenomenon is common with phytoestrogen [50]. In addition, belutin a triterpernoid, structurally related to 2 and 3 have shown estrogenic activity in MCF-7 cells [51]. It is noteworthy that 2 has osteogenic properties in vitro [52]; which can account for beneficial effects of *M. macrophylla* in postmenopausal osteoporosis.

Besides their estrogenic properties, phytoestrogens exert a wide variety of pharmacological effects including cytotoxicity [10]. Estrogen receptors are one of the targets in anti-breast cancer therapy. For this reason the cytotoxic potential of compounds from *M. macrophylla* was assessed. It was noted that 2 induced cytotoxic effects on MCF-7 (CC_50_ = 110 μM) and MDA-MB-231 (CC_50_ = 160 μM) cells. This result is in accordance with some reports [53, 54]. Weak cytotoxic effects were also observed with 6, 7, 8, 13 and PF. These results are consistent with some reports which postulate that phytoestrogens may exert two opposite actions depending on their concentrations [55]. At lower concentrations (<10 μM), some phytoestrogens, like genistein, stimulate growth of ER positive MCF-7 cells, but not the ER negative MDA-MB-231 breast cancer cells. At higher concentrations survival of both types of breast cancer cells decreases. It was postulate that at the lower concentration, phytoestrogens acted as ligands of ERs and stimulate metabolic pathways, which in turn induced cell proliferation. However, at higher concentrations, mechanisms which are not dependent on ER pathway such as antioxidant properties of the flavonoids seem to be triggered [55]. The above arguments might also explain why MDA-MB-231 cells were found most sensitive than MCF-7 cells to *M. macrophylla* isolates in this study; they could induce their cytotoxicity by a non-dependent ER pathway.

The E-screen assay measures the direct estrogenicity at the target cell level whereas, in animal studies there are a complexity of several systems and processes involved, including metabolism and clearance. Hence, in this study, the phenolic fraction of *M. macrophylla* has been tested in vivo as a whole of all the isolates. Estrogenic effects of *M. macrophylla* phenolic fraction were assessed in vitro and in vivo. In vitro it can be seen that phenolic fraction exhibited estrogenic activity but this effect seems to be lower than the ones induced by pure compounds. This can be explained by the phenomenon of the extract dynamic and molecules interactions [56]. As for the pure compounds, the MCF-7 cells proliferation induced by phenolic fraction of *M. macrophylla* decreased with increasing concentrations. It has also been observed that the ability of the *M. macrophylla* phenolic fraction to transactivate ERs in reporter gene assay change with concentration. However, the *M. macrophylla* phenolic fraction induced a most potent transactivation of ERα as well as ERβ at the lower concentrations (10^−3^ and 10^−2^ μg/mL) than that previously reported with methanol crude extract (10^−1^ and 10^1^ μg/mL) [18]. The in vivo estrogenic activity of this fraction was assessed in an uterotrophic assay. Phenolic fraction at the dose of 10 mg/kg induced a significant increase in uterine wet weight, uterine total protein level, vaginal and uterine epithelial height as well as in mammary glands differentiation. These results confirm those previously obtained with *M. macrophylla* methanol extract on estrogen target organs [18]. However, as observed in reporter gene assay, the obtained results indicate that the phenolic fraction is more efficient than the crude methanol extract. We can hypothesized that 6, 13, 10, 11, 3 as well as others potential active principles detected in *M. macrophylla* phenolic fraction are able to transactivate the ERα as observed in the reported gene assay and trigger proliferation of vaginal and uterine epithelial cells as it did in MCF-7 cells in vitro. Such effects have been reported to be mediated by ERα [57]. Cell proliferation is followed by protein synthesis increased; this can explain the observed increased of total protein level in uterus after 3 consecutive days of treatment. Authors report that this increase in protein amounts into cells is responsible for the uterine wet imbibition [58]. Conversely, the increase in the acini size of mammary gland observed in vivo with the *M. macrophylla* phenolic fraction, can be explained by the capacity of this fraction to induce MCF-7 cells proliferation, which are adenocarcinoma cells expressing ERα. Further, PF induced a weak cytotoxic activity in both MCF-7 and MDA-MB-231 cells. This result can be a promising search of anti-breast cancer therapy.

The most common concern in postmenopausal women is hot flushes which are experienced by as many as 75 % of menopausal women [59]. It is well documented that the effectiveness of xenobiotic in compensating the effects of estrogen deprivation can be assessed by its ability to alleviate hot flushes [60]. It appears from the analysis of core temperatures that, the average duration and frequency of hot flushes was significantly reduced in the group treated with the phenolic fraction at the dose of 10 mg/kg. All these results suggest that this fraction contains secondary metabolites with estrogenic activity which can reverse the thermoregulatory dysfunction related to post-oophorectomy depletion of endogenous estrogens. Indeed, Freedman et al. [59] reported that estrogen and estrogenic substances raise the sweating threshold and expand the thermoneutral area in postmenopausal symptomatic women.

## Conclusion

*Millettia macrophylla* has estrogenic properties, given its high amount of flavonoids as well as triterpernoids mainly, variabilin (13), aformorsin (7), pisatin (10), stigmastenone (3), flemichaparin B (11), formononetin (6), lupeol (2), which exhibited estrogenic effects in this work. 11, 3 and 2 have been described for the first time as estrogenic in this work and together with 7, 13, 10 and 6 could be the active principles of *M. macrophylla*. The phenolic fraction prepared from methanol extract of *M. macrophylla* appears to be more efficient than the methanol crude extract. It could be a good candidate for the preparation of an improved traditional medicine from *M. macrophylla* able to alleviate some menopausal complaints such as vaginal dryness and hot flushes. However, further studies have to be developed to achieve a better understanding concerning the interactions between the ligand-receptor binding of these compounds and ERs.

## Additional file

Additional file 1:
**Figure S1.** 1H NMR (600 MHz, CD3OD) spectrum of compound 6. **Figure S2.** ESI-MS (negative mode ionization) spectrum of compound 6. **Figure S3.** 1H NMR (600 MHz, CD3OD) spectrum of compound 13. **Figure S4.** ESI-MS (positive mode ionization) spectrum of compound 13. (DOC 112 kb)

## Abbreviations

C: Compound
CC_50_: Cytotoxic concentration for 50% of cells
C-hex: Cyclo-hexane
CNH: Cameroon national herbarium
DCM: Dichloromethane
DMEM: Dulbecco’s modified eagle medium
DMSO: Dimethylsulfoxide
ER: Estrogen receptor
EtOAc: Ethylacetate
EtOH: Ethanol
F: Fraction
FBS: Fetal bovine serum
FCS: Fetal calf serum
HEPES: 4-(2-hydroxyethyl)-1-piperazine ethane sulfonic acid
HPLC: High performance liquid chromatography
MeOH: Methanol
MPLC: Medium pressure liquid chromatography
NMR: Nuclear magnetic resonance
PDA: Photodiode array
PE: Proliferative effect
PF: Phenolic fraction
RP-18: Reverse phase 18
RPE: Relative proliferative effect
RPMI: Roswell Park Memorial Institute
SRB: Sulforhodamine-B
TLC: Thin layer chromatography
UHPLC: Ultra high performance liquid chromatography
UV–vis: Ultraviolet visible
