# Supplementary material for: Estrogenic and cytotoxic potentials of compounds isolated from Millettia macrophylla Benth (Fabaceae): towards a better understanding of its underlying mechanisms
Source: BMC Complement Altern Med. 2016 Oct 26;16:421. doi: 10.1186/s12906-016-1385-5 (PMC5396542; doi:10.1186/s12906-016-1385-5)
Supplement: Additional file 1: — Figure S1. 1H NMR (600 MHz, CD3OD) spectrum of compound 6. Figure S2. ESI-MS (negative mode ionization) spectrum of compound 6. Figure S3. 1H NMR (600 MHz, CD3OD) spectrum of compound 13. Figure S4. ESI-MS (positive mode ionization) spectrum of compound 13. (DOC 112 kb) [file 12906_2016_1385_MOESM1_ESM.doc]

Supporting Information

**Estrogenic and cytotoxic potentials of compounds isolated from *Millettia macrophylla* Benth. (Fabaceae): towards a better understanding of its underlying mechanisms**

Stéphane Zingue^1,2,3,4^,*, Job Tchoumtchoua^2,3^, Dieudonnée Mireille Ntsa^2^, Louis Pergaud Sandjo^4^, Julia Cisilotto^4^, Chantal Beatrice Magne Nde^5^, Evelyn Winter^4^, Charline Florence Awounfack^2^, Derek Tantoh Ndinteh^6^, Colin Clyne^5^, Dieudonné Njamen^2,6^, Maria Halabalaki^3^, Tânia Beatriz Creczynski-Pasa^4^,*

*^1^ Department of Life and Earth Sciences, Higher Teachers’ Training College, University of Maroua, P.O. Box 55 Maroua, Cameroon*

*^2^ Department of Animal Biology and Physiology, Faculty of Sciences, University of Yaoundé I, P.O. Box 812 Yaoundé, Cameroon*

*^3^ Division of Pharmacognosy and Natural Products Chemistry, School of Pharmacy, University of Athens, Panepistimioupoli Zografou 15771, Athens, Greece*

*^4^ Department of Pharmaceutical Sciences, Health Sciences Centre, Federal University of Santa Catarina, CEP 88040-900, Florianópolis, Santa Catarina, Brazil*

*¶^5^ Hudson Institute of Medical Research, Clayton, Victoria 3168, Australia*

*^6^ Department of Applied Chemistry, Faculty of Sciences, University of Johannesburg, Doornfontein 2028, South Africa*

*Corresponding authors: Tânia Beatriz Creczynski-Pasa, Group of interaction micro and macromolecular studies, Department of Pharmaceutical Sciences, Health Sciences Centre, Federal University of Santa Catarina, CEP 88040-900, Florianópolis, Santa Catarina, Brazil. Phone: +55 4837212212; Email: [tania.pasa@ufsc.br](mailto:tania.pasa@ufsc.br). Stéphane Zingue, Laboratory of Physiology and Natural Products Research, Department of Live and Earth Sciences, Higher Teachers’ Training College, University of Maroua, P.O. Box 55, Maroua, Cameroon. Phone: +237-677 272 033; Email: [stephanezingue@gmail.com](mailto:stephanezingue@gmail.com)


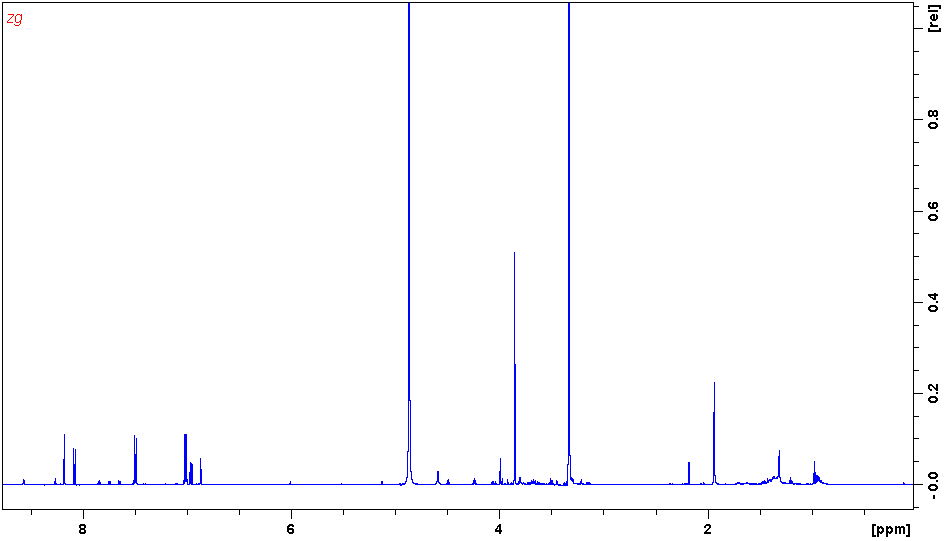


Fig. S1 ^1^H NMR (600 MHz, CD_3_OD) spectrum of compound **6**

Fig. S2 ESI-MS (negative mode ionization) spectrum of compound **6**


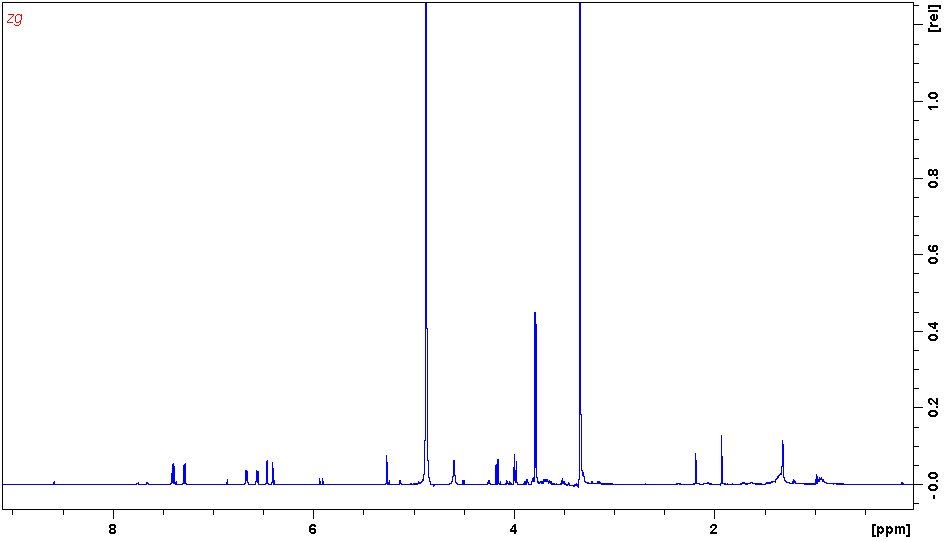


Fig. S3 ^1^H NMR (600 MHz, CD_3_OD) spectrum of compound **13**


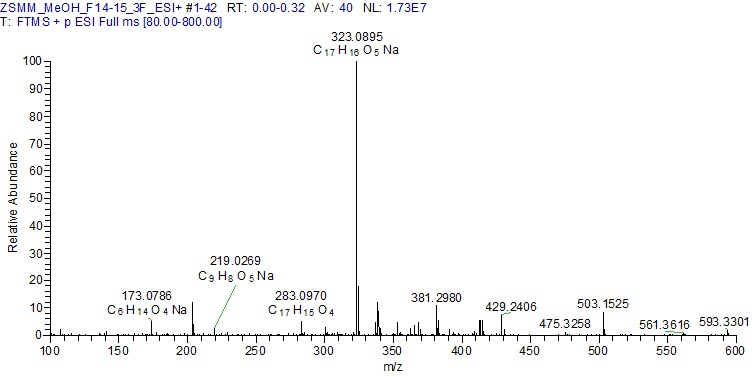


Fig. S4 ESI-MS (positive mode ionization) spectrum of compound **13**
